# Early Social Enrichment Modulates Tumor Progression and p53 Expression in Adult Mice

**DOI:** 10.3390/biom12040532

**Published:** 2022-03-31

**Authors:** Silvia Middei, Ludovica Giorgini, Valentina Vacca, Francesca Storri, Sabrina Putti, Georgios Strimpakos, Marcello Raspa, Ferdinando Scavizzi, Fabiola Moretti, Francesca R. D’Amato

**Affiliations:** 1 Institute Biochemistry and Cell Biology, National Research Council of Italy (CNR), 00015 Monterotondo, Italy; ludovica.giorgini@unicatt.it (L.G.); valentina.vacca@outlook.it (V.V.); francesca_vt@hotmail.it (F.S.); sabrina.putti@cnr.it (S.P.); georgios.strimpakos@cnr.it (G.S.); marcello.raspa@cnr.it (M.R.); fscavizzi@emma.cnr.it (F.S.); fabiola.moretti@cnr.it (F.M.); francesca.damato@cnr.it (F.R.D.); 2 European Brain Research Institute, 00161 Rome, Italy; 3 Department of Translational Medicine and Surgery, PhD Course in Nutrition, Metabolism, Aging and Gender Medicine, Catholic University “Sacred Heart”, 00168 Rome, Italy; 4 EMMA/Infrafrontier/IMPC, 00015 Monterotondo, Italy

**Keywords:** social enrichment, tumorigenesis, p53, DNA-damage, 3-methylcolantrene (3MCA), fibrosarcoma, p21

## Abstract

Epidemiological evidence indicates that stress and aversive psychological conditions can affect cancer progression, while well-being protects against it. Although a large set of studies have addressed the impact of stress on cancer, not much is known about the mechanisms that protect from cancer in healthy psychological conditions. C57BL/6J mouse pups were exposed to an environmental enrichment condition consisting of being raised until weaning by the biological lactating mother plus a non-lactating virgin female (LnL = Lactating and non-Lactating mothers). The Control group consisted of mice raised by a single lactating mother (L = Lactating). Four months after weaning, mice from LnL and L conditions were exposed to intramuscular injection of 3-methylcolantrene (3MCA), a potent tumorigenic drug, and onset and progression of 3MCA-induced fibrosarcomas were monitored over time. Pups from the LnL compared to the L group received more parental care and were more resilient to stressful events during the first week of life. In association, the onset of tumors in LnL adults was significantly delayed. At the molecular level, we observed increased levels of wild-type p53 protein in tumor samples of LnL compared to L adults and higher levels of its target p21 in healthy muscles of LnL mice compared to the L group, supporting the hypothesis of potential involvement of p53 in tumor development. Our study sustains the model that early life care protects against tumor susceptibility.

## 1. Introduction

Epidemiological evidence hints that stress negatively impacts cancer progression while social interactions and positive psychological conditions can ameliorate prognosis in tumor patients [1,2]. Animal studies proved the effects of stress on tumor progression and have been instrumental in delineating the underlying molecular mechanisms (reviewed in [3]). These include activation of the neuroendocrine stress response, release of glucocorticoids, and suppression of immune functions, which have been acknowledged in patients with cancer [1,3,4]. On the other hand, some clinical evidence indicates that psychological and social support ameliorate immune response and may also increase survival in cancer patients [2,5,6]. However, clinical reports are sometimes mixed [7]. Furthermore, systematic studies are still missing [3], and much remains to be elucidated about the biological mechanisms that promote the positive effects of psychosocial support interventions [8].

The process of malignant transformation universally involved genetic damage and oncogenic signaling. TP53 is one of the most relevant tumor suppressor genes both in humans and mice. It prevents cancer by promoting cell cycle arrest or cell death of transformed cells through different signaling pathways [9]. In humans, a vast proportion of tumors are associated with missense mutations of the p53 (TP53) gene, which leads to loss of p53 oncosuppressive function [10]. In mice, loss of p53 alleles (p53^−/−^) predisposes to different spontaneous tumors, among which fibrosarcomas, in about 83% of animals [11].

Conversely, the presence of one supernumerary p53 allele delays the onset of experimentally induced tumors [12]. Emerging evidence indicates that p53 is also involved in the homeostatic regulation of immune responses [13], and its expression can be negatively regulated by factors associated with psychological stress. In hepatocarcinoma cells, the stress hormone cortisol has been associated with lower levels of p53 [14]. In mice, restraint stress increases glucocorticoids levels, which reduce p53 expression, thereby promoting the growth of xenograft tumors in a p53-dependent manner [15]. At present, a link of p53 oncosuppressive activity with a positive psychosocial environment has not been evidenced.

Here, we analyzed the effect of an enriched psychosocial environment on tumor development and whether p53 is involved. To this purpose, we combined a behavioral protocol of early social enrichment in mice with the DNA damage-induced tumorigenesis. Our results indicate that early social enrichment delays induced fibrosarcomas, and this is associated with increased p53 activity.

## 2. Materials and Methods

### 2.1. Animals, Social Enrichment, and Experimental Groups

C57BL/6J mice were obtained from CNR EMMA-INFRAFRONTIER-IMPC, “A. Buzzati-Traverso” International Campus, Monterotondo Scalo. Animals were housed with food and water ad libitum at 21 ± 1 °C and with a 12 h light/dark cycle. Breeding groups (one male, two littermate females) of 2-month-old C57BL/6J mice were kept together for two weeks. A few days before parturition, female mice were isolated (control condition, L = Lactating) or caged with a same-age unfamiliar virgin C57BL/6J female (social enrichment condition, LnL = Lactating, and non-Lactating). Three cohorts of female mice were used, generating all the mice used in different experiments. Housing condition and room temperature were constant with a 12 h:12 h light:dark cycle. A standard amount of bedding and nest material (soft paper) were provided for each cage.

Around the delivery day, cages were inspected twice per day to check for newborn pups, in which case day 0 was set. On postnatal day 1 (PND1), pups were counted, leaving unchanged the number of pups in each litter. Litters with less than four pups were not included in the experiments. Bedding in the cages was not cleaned until PND10 and mother–pups interaction was assessed from PND1 to PND8.

Male and female mouse pups from each litter were raised together in the L or LnL condition. However, all the experiments were conducted on male mice only in order to avoid interference due to hormonal cycles in females. On PND8 male pups were weighed and evaluated for body temperature and ultrasonic vocalizations (USVs). Concurrently, biological mothers were probed for anxiety levels in the Elevated Plus Maze (EPM) apparatus.

Upon weaning at PND28, male mice were caged in groups of four made of two pairs of siblings from two distinct home-cages in order to ensure the same variability within each cage. From a first group of mice peripheral blood was collected for assessment of T lymphocytes basal state at 2 months of age.

**Figure 1:**
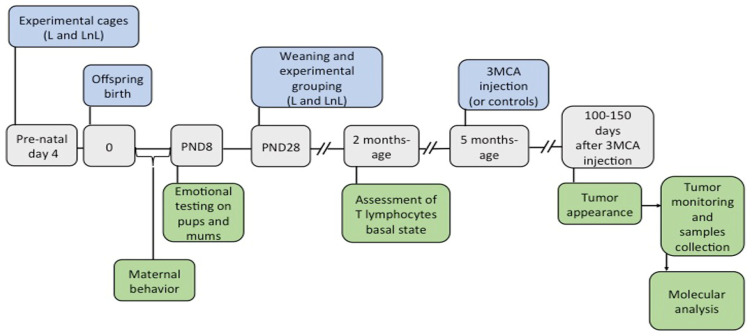
Experimental design and groups. **Top panels**: experimental manipulations are shown in blue and include: 1. grouping pregnant females in L and LnL cages four days before offspring birth; 2. weaning at postnatal day 28 (PND28) during which male mice were caged in groups of four made of two pairs of siblings from two distinct L or LnL home-cages; 3. 3-methylcolantrene injection in the right rear leg of 8 L and 8 LnL mice (the same number of mice from the two groups were left undisturbed and used as controls). **Middle panels:** temporal timeline for the experiments, with gray boxes indicating key time points. **Bottom panels:** measured variables are reported in green boxes and include: 1. assessment of maternal behavior during the first week of pups’ life; 2. pups’ ultrasonic vocalizations and anxiety behavior measured on biological mothers on the elevated plus maze on PND8; 3. assessment of T lymphocytes basal state; 4. tumor monitoring from their onset until sacrifice; 5. molecular analysis on tumor and muscle samples.

A second group of mice was split into two sub-groups used respectively for carcinogenesis experiments and controls. At five months of age, the experimental sub-group of mice was subjected to carcinogenesis induction and sacrificed at the occurrence of fibrosarcomas. Mice from the control sub-group, which were not exposed to carcinogenesis induction, were sacrificed in parallel for tissue collection for molecular investigation in basal conditions. The simplified scheme in Figure 1 provides an overview of the experimental design.

### 2.2. Behavioral Tests and Measurement of Body Temperature

Mother–offspring interaction was assessed in the animal room, leaving the cages undisturbed during the first week of pups’ life. Evaluations were performed by measuring the number of sampling points (32 in total, one point every 2 min over 30 min observation, twice a day) in which the following behaviors were observed: 1. active maternal care (nursing posture, grooming, licking, nest building) by adult female/s; 2. female/s out of the nest (litters left alone). For the LnL group, these specific behaviors were assessed regardless of female identity (lactating or non-lactating).

On PND8, pups were transiently separated from adult females in order to assess both pups’ response to separation and anxiety levels in mothers.

Pups’ response to separation was assessed by analyzing ultrasonic vocalizations (USVs) emitted by a sub-group of pups (*n* = 9 L and *n* = 8 LnL) during 5 min intervals. To this aim, mothers were removed from the home cage and 5 min later one pup at a time was taken from the nest and confined in a clean bedding box at room temperature equipped with an ultrasound microphone and dedicated USV recording and analysis software (Avisoft Bioacoustics Technology, Berlin, Germany). Body temperature was measured immediately before and after the 5-min USVs assessment by keeping an infra-red thermometer (Bioseb) 1 cm above the interscapular region [16]. To avoid excessive isolation, no more than three pups per litter were tested. Pups not used for USVs (*n* = 11 L and *n* = 10 LnL mice) were left together in the nest, and their body temperature and body weight were recorded after 30 min of separation from the adult females. Anxiety levels were assessed in biological mothers only (*n* = 11 L and *n* = 10 LnL) by using an elevated plus-maze equipped with two closed and two open arms. The percent of time spent in open arms over 5 min was measured and used as an index of low anxiety.

### 2.3. Chemical Carcinogenesis and Tissue Collection

Fibrosarcomas were induced in 5-month-old mice from L and LnL groups (*n* = 8/group) by intramuscular injection of 0.5 mg/100 µL of 3-methylcolantrene (3MCA, Sigma) in the right rear leg. 3MCA was dissolved in sesame oil at a concentration of 5 mg/mL. After about three months post-injection, the tumor mass became palpable. Mice were examined twice a week for tumor development and size, using a caliper at the indicated time points. Tumor size was calculated as the product of two perpendicular diameters (mm). The animals were euthanized for ethical reasons when the tumor reached a weight of about 3 gr or when the animals showed signs of suffering. Euthanasia occurred by saturated room with CO_2_ saturation levels greater than 70%.

Upon sacrifice, fibrosarcomas isolated from the muscle were measured, minced, immediately frozen in liquid nitrogen, and stored at −80 °C. In parallel, *n* = 8 L and *n* = 8 LnL control mice (non-injected with 3MCA) were euthanized as above for right rear leg muscle collection.

### 2.4. Protein Analysis

For WCEs, tissues were lysed with RIPA buffer (50 mMTris–Cl, pH 7.5, 150 mMNaCl, 1% NP-40, 0.5% Na deoxycholate, 0.1% SDS, 1 mM EDTA) supplemented with a cocktail of protease inhibitors (Roche, Basilea, CH, Switzerland).

For Western blot, all SDS–PAGE were transferred onto PVDF membranes (Millipore, Burlington, MA, USA). Membranes were developed using the enhanced chemiluminescence (ECL Amersham, Little Chalfont, UK and Cyanagen, Bologna, Italy) by the chemiluminescence imaging system Alliance 2.7 (Uvitec, Cambridge, UK) and quantified by the software Alliance V_1607.

The following primary antibodies were used: anti-p53 FL-393 1:1000 (#sc- 6243 Santa Cruz Biotechnology Inc., Dallas, TX, USA), anti-p53 1C12 1:1000 (#2524 Cell Signaling Technology Inc, Danvers, MA, USA), anti-p21 F-5 1:1500 (#sc- 6246 Santa Cruz Biotechnology Inc., Dallas, TX, USA), α-GAPDH 1:8000 (#GA1R, ThermoFisher, Waltham, MA, USA), HRP α-goat 1:8000 (#Sc-2768 Santa Cruz Biotechnology Inc., Dallas, TX, USA), HRP α-mouse 1:5000 (Bio-Rad, Hercules, CA, USA), anti-Bax N-20 1:1000 (#sc-493 Santa Cruz Biotechnology Inc., Dallas, TX, USA), anti-Mdm2 mix of clone Ab1 1:1000 (#OP46, Merck KGaA, Darmstadt, Germany) and 2A10 1:1000 for human Mdm2 (#MABE281, Merck KGaA, Darmstadt, Germany).

### 2.5. mRNA Analysis

#### 2.5.1. RNA Extraction and cDNA Synthesis

Total RNA was isolated from both muscle and fibrosarcoma samples using Trizol Reagent (Thermofisher, Newtown, CT, USA) following the manufacturer’s instructions. We then obtained cDNA by in vitro retrotranscription using Ultrapure SMART MMLV Reverse Trascriptase (Takara Bio, San Jose, CA, USA).

#### 2.5.2. Genetic Analysis of TP53 Mutation

Analysis of tp53 mutation in fibrosarcomas was performed by sequencing p53 cDNA. The exons 4, 5, 6, 7, 8, 9, and 10 of p53 gene, in which reside the hot spot mutations, were amplified by polymerase-chain-reaction (PCR) with the use of AmpliTaq Gold (Applied Biosystems, Newtown, CT, USA) and following primer sets: 2F: 5′-TGTCATCTTTTGTCCCTTCTC-3′; 2R: 5′-GTGATGATGGTAAGGATAGGT-3′; 3F: 5′-TACATGTGTAATAGCTCCTGC-3′; 3R: 5′-TCAGCCCTGAAGTCATAAG-3′. Purified PCR products were sequenced by external service (Eurofins Genomics, EbersbergDE). Sequence data were analyzed by means of Chromas Technelysium DNA sequencing software followed by a manual review.

#### 2.5.3. RT-PCR

Real-time PCR was performed on cDNA using an ABI 7300 Real-Time PCR System with 7300 System SDS Software (ThermoFisher, Newtown, CT, USA) using SensiMix SYBR Low-ROX Mix (Bioline, London, UK). The detection of a single amplicon was verified using a dissociation curve. Normalized, relative mRNA levels were calculated according to the ΔΔCt method, using endogenous reference gene for normalization. Primers efficiencies were calculated from a dilution curve and determined to be within the acceptable range of 90–110% efficiency. Mouse p21 forward: 5′-GCAGATCCACAGCGATATCCA-3′ and p21 reverse: 5′-AGACAACGGCACACTTTGCTC-3; mouse β actin forward: 5′-CGA TGC CCT GAG GCT CTT T-3′ and mouse β actin reverse: 5′-TAGTTTCATGCATGCCACAGGAT-3.

### 2.6. FACS Analysis

Blood samples were collected from tail vein of 2 months old mice (*n* = 7 L and *n* = 7 LnL) into EDTA coated tubes. Whole blood was treated with Lysis buffer (BD) to eliminate erythrocytes before adding fluorochrome-labeled antibodies to bind specifically to leukocyte surface antigens. Cells were analyzed by flow cytometry (BD FACS CAntoII, BD Biosciences Becton Drive Franklin Lakes, NJ, USA) using combinations of the following monoclonal antibodies: CD45 BV510 (563891 BD), CD4 PECY7 (552775 BD), CD8a BB515 (584422 BD), CD11b APC (553312 BD), CD3 BB700 (566494 BD), CD19 PE (557399 BD). 10,000 events were acquired and stored for each analysis.

Lymphocytes were identified by their characteristic appearance on a dot plot of FSC versus SSC. Fluorescence overlap was then compensated electronically using lymphocytes stained with single colors. Data were analyzed using FlowJo after singlet doublet discrimination and live cells (Fixable viability stain 700 BD bioscience). Leukocytes were identified with SSC and CD45 gates. All populations were identified as subpopulation of CD45 positive cells and reported as percentage of leukocytes.

## 3. Results

### 3.1. LnL Mice Receive More Maternal Care Than L Mice during the First Postnatal Week

**Figure 2:**
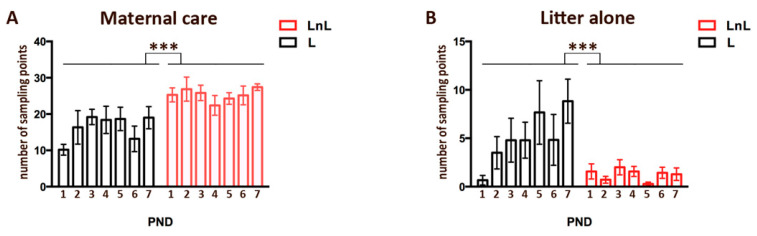
Increased maternal care from LnL mothers during the first postnatal week: (**A**) number of sampling points (mean ± SEM) for active maternal care (nursing posture, grooming, licking, nest building) during daily monitoring (*** *p* <0.0001; two-way Anova for repeated measures with between factors being L and LnL groups and days being within measures); (**B**) number of sampling points (mean ± SEM) for litter left alone during daily monitoring. (*** *p* <0.0001; two-way Anova for repeated measures with between factors being L and LnL groups and days being within measures). Daily monitoring consisted of 32 recordings collected during 30-min sessions repeated twice over 7 days. PND = post-natal day.

For social enrichment, we used the double-mothering model [17,18,19]. Briefly, four days before parturition, pregnant female mice were isolated (control condition, L = Lactating) or caged with a same-age unfamiliar virgin C57BL/6J female (social enrichment condition, LnL = Lactating plus non-Lactating) (Figure 1). Mother–offspring interaction was assessed during the first week of pups’ life by monitoring active parental care and time spent out of the nest by the adult females. The number of intervals of active maternal care was higher in LnL vs L females (group effect: F_1,11_ = 78.81, *p* < 0.0001) regardless of the PND (day effect: F_6,66_ = 0.89, n.s.: group x day interaction: F_6,66_ = 0.93, n.s.; Figure 2A). The number of intervals in which pups were alone in the nest was higher in L vs. LnL litters (F_1,11_ = 28.88, *p* < 0.0001, Figure 2B). Overall, these data indicate that pups raised by two adult females receive more maternal care compared to the control L group.

### 3.2. LnL Condition Favors Resilience to Separation in Pups and Reduces Anxiety in Mothers

Raising mice in a social enrichment condition has a positive impact on both dams and pups [17,18]. Therefore, we wondered whether the enriched social environment derived from LnL condition affects the stress response of animals. To answer this question, on postnatal day 8 (PND8), we temporarily separated litters from mothers and assessed pups’ response to isolation and biological mothers’ anxiety levels.

**Figure 3:**
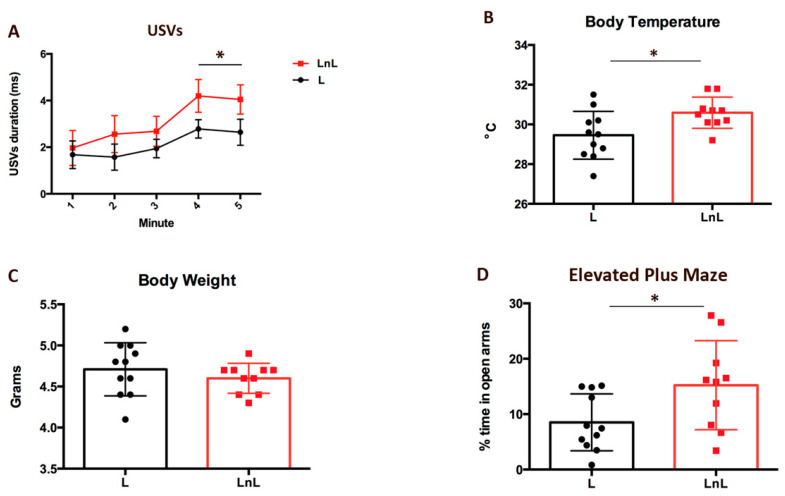
LnL condition enhances resilience in 8-day-old pups and reduces anxiety in biological mothers. (**A**) Duration (mean ± SEM) of ultrasonic vocalizations (USVs) emitted from L and LnL pups during 5 min isolation. (* *p* = 0.05, one-way Anova for repeated measures, with time as within factor); (**B**) body temperature (mean ± SEM) of L and LnL pups after 20 min isolation (* *p* < 0.05, two-tailed unpaired *t*-test). (**C**) body weight (mean ± SEM) of L and LnL pups (n.s.; two-tailed unpaired *t*-test) (**D**) percentage of time spent in open arms (mean ± SEM) of an elevated plus maze by biological mothers from L and LnL groups (* *p* < 0.05; two-tailed unpaired *t*-test).

To assess pups’ response to maternal separation, we measured USVs emission after separation from adult females. For both L and LnL mice, time spent in vocalizing over the 5 min recording progressively increased with a peak at the last 2 min (F_1,4_ = 39.308, *p* = 0.0003, Figure 3A). However, the total duration of USVs emitted during the last 2 min was longer in pups of LnL compared to those of the L group (t_(15)_ = −2.123, *p* = 0.058, Figure 3A).

In addition, pups’ body temperature was comparable between groups before USVs (30.01 ± 0.25 °C in L mice and 29.98 ± 0.21 °C in LnL mice, t_(24)_ = 0.106; *p* = 0.916, n.s.) and dropped down similarly during the 5 min interval for USVs (28.55 ± 0.29 °C in L mice; from to 28.50 ± 0.20 °C in LnL mice, t_(24)_ = 0.122; *p* = 0.903, n.s.). However, body temperature collected in pups 30 min after separation from adults but kept together in the nest was significantly higher in LnL than L mice (t_(19)_ = 2.53; *p* = 0.0203; Figure 3B). Since body weight was also comparable in mice from the two groups (t_(19)_ = 0.937; *p* = 0.3605, n.s.; Figure 3C), higher body temperature in LnL-grouped mice denoted better thermoregulation which is indicative of enhanced defense response to mother-isolation stress [20]. Furthermore, we assessed the levels of modulators of inflammatory response T lymphocytes in peripheral blood in mice at 2 months of age. This analysis indicated that levels of T lymphocytes (including CD4+ and CD8+ T lymphocytes) were comparable between mice from L and LnL groups (Figure S1), thereby excluding any difference in their basal inflammatory status.

To further assess the effect of social enrichment, we compared anxiety levels in biological mothers from L and LnL groups.

Anxiety in biological mothers was assessed by measuring their preference for closed vs open arms of the elevated plus maze. Although the general locomotor activity measured by the number of entries in open and closed arms was comparable between biological mothers from the L and LnL group (for L = 18.82 ± 3 for LnL = 19 ± 2; t_(19)_ = 0.0406, n.s.), the percentage of time spent in open arms was significantly higher in the LnL compared to the L group (t_(19)_ = 2.298, *p* = 0.033, Figure 3D), indicating that biological mothers from LnL group are less anxious than L mothers.

Overall, the above data indicate that pups raised in the LnL condition are more reactive and resilient to the effects of stress conditions with respect to mice raised in standard L condition. Furthermore, biological mothers that raise their pups with one other female in the LnL condition are less anxious than biological mothers in the L condition, likely contributing to the enhanced resilience of LnL pups.

### 3.3. LnL Condition Delays the Onset of 3MCA-Induced Fibrosarcomas in Adult Mice

Stress is associated with increased cancer progression [3]. Conversely, a supportive psychological environment can enhance endogenous responses that contrast cancer progression [5]. We therefore challenged mice raised in LnL and L conditions with DNA damage-induced tumorigenesis. Since the LnL condition confers important improvements in several cognitive and social parameters during adulthood [17,18,19], we probed the impact of LnL enrichment on tumor development in the adult life.

**Figure 4:**
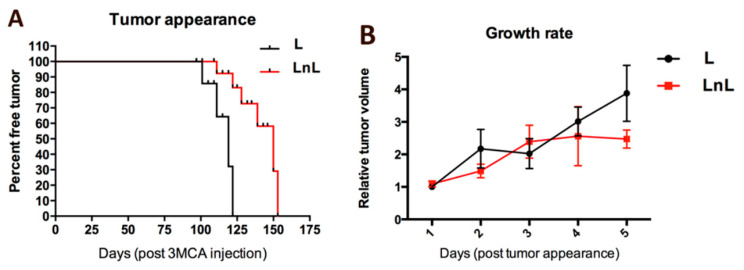
Development of fibrosarcomas is delayed in mice from the LnL group. (**A**) Curves reporting the appearance of palpable tumors from L and LnL mice. (*p* < 0.01; χ^2^ test); (**B**) relative tumor volume (mean ± SEM) over the interval of time between tumor appearance and mouse sacrifice (n.s., Mann–Whitney U test for each day).

We injected the right rear legs of 5-months mice with 3MCA, a potent tumorigenic agent [12], and monitored the development of fibrosarcomas. A significant delay in the appearance of 3MCA-induced fibrosarcomas was observed in mice raised in LnL condition compared to L controls (χ^2^ = 7.984, *p* = 0.0047; Figure 4A). Conversely, the growth rate of those tumors was not different between the two groups (Mann–Whitney U test for L vs LnL each day comparisons, n.s.; Figure 4B).

These data indicate that social enrichment during the first weeks of life is a condition sufficient to reduce the sensitivity to DNA damage-induced tumorigenesis.

### 3.4. LnL Condition Is Associated to Increased p53 Activity

**Figure 5:**
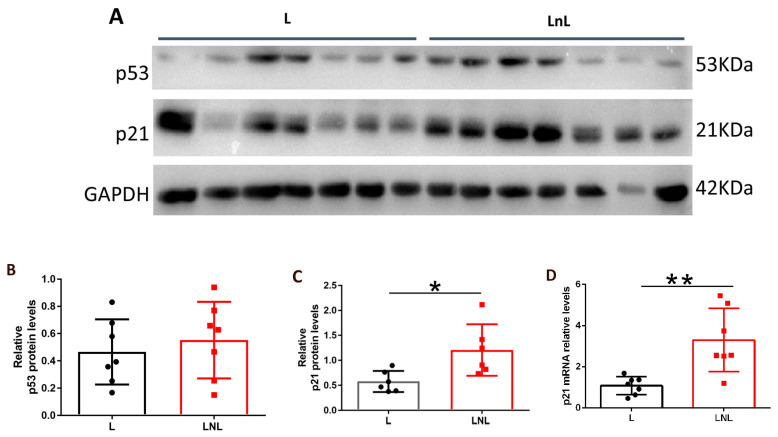
P53 activity is increased in LnL group. (**A**) WB analysis of indicated protein levels in muscles derived from animals of L and LnL groups, GAPDH was used as loading control. (**B**) Densitometric analysis of p53 relative to GAPDH levels (mean ± SD) in indicated groups (n.s., two-tailed unpaired *t*-test, *n* = 7/group). (**C**) Densitometric analysis of p21 relative to GAPDH levels (mean ± SD) in indicated groups (* *p* < 0.05, two-tailed unpaired *t*-test, *n* = 7/group). (**D**) RT-PCR analysis of p21 mRNA levels in indicated groups (** *p* < 0.01, two-tailed unpaired *t*-test, *n* = 7/group).

P53 is a master regulator of the cellular response to DNA damage, and its levels affect the development of 3MCA-induced fibrosarcomas [12,21]. Therefore, we wondered whether altered levels and/or activity of p53 are associated with delayed fibrosarcoma development in the LnL group. We first analyzed p53 in the healthy muscle of mice from L and LnL condition. Analysis of whole-cell extracts (WCE) of muscle of control L and LnL animals did not reveal significant different protein levels of p53 (t_(12)_ = 0.6234, n.s.) (Figure 5A,B). Conversely, levels of one of its main targets, p21/WAF1, were significantly increased in LnL compared to L group (t_(10)_ = 2.762, *p* = 0.0201) (Figure 5A,C). Accordingly, levels of p21 mRNA were significantly increased (t_(12)_ = 3.679, *p* = 0.0032) (Figure 5A), supporting the hypothesis of increased activity of p53 in that group.

**Figure 6:**
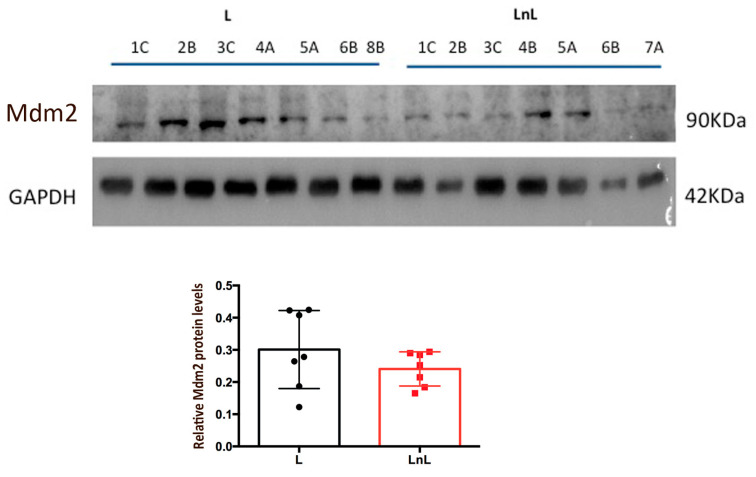
Lower Mdm2 levels in LnL mice. (**Top**) WB analysis of indicated protein levels in muscles derived from animals of L and LnL groups, GAPDH was used as loading control. (**Bottom**) Graphs reporting densitometric analysis of Mdm2 relative to GAPDH levels (mean ± SD) in indicated groups (n.s., two-tailed unpaired *t*-test, *n* = 7/group).

To further confirm this data, we observed decreased, although not significant, levels of Mdm2 (Figure 6), the main p53 inhibitor, in the LnL compared to the L group, supporting the hypothesis of a reduced inhibition of p53 activity. Accordingly, a rough correlation between the levels of the two proteins was observed (Figure S2). Analysis of other p53 targets as BAX (BCL2 Associated X, Apoptosis Regulator) did not reveal significant alterations (Figure S3).

**Figure 7:**
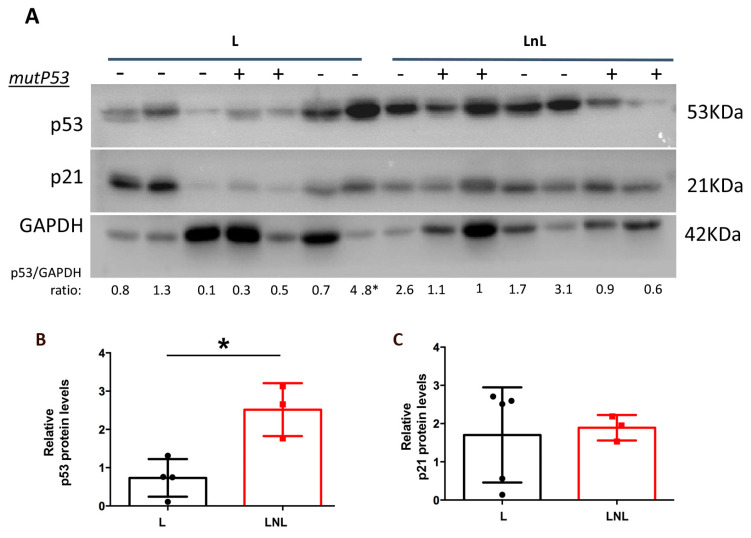
P53 levels are increased in fibrosarcoma of LnL animals. (**A**) WB analysis of indicated protein levels in fibrosarcoma derived from animals of L and LnL groups, GAPDH was used as loading control. Numbers under the lanes report the densitometric value of p53/GAPDH ratio. * marks an outlier value (by ROUT method, Q = 0.1%) not shown in the graph. (**B**) Densitometric analysis of wild type p53 relative to GAPDH levels (mean ± SD) in indicated groups (* *p* < 0.05, two-tailed unpaired *t*-test, *n* = 4 L, 3 LnL). (**C**) Densitometric analysis of p21 (from samples expressing wild type p53) relative to GAPDH levels (mean ± SD) in indicated groups (n.s., two-tailed unpaired *t*-test, *n* = 5 L, 3 LnL).

Upon DNA damage, p53 levels are increased, and its oncosuppressive activity is activated. Indeed, analysis of 3-MCA-induced tumor samples revealed increased levels of p53 in fibrosarcoma of LnL compared to L group (Figure 7A and Figure S4). In tumors, p53 levels may also be increased due to the stabilization of mutant p53 protein. To ascertain whether increased p53 levels in the LnL group are associated or not with the presence of mutant proteins, we sequenced p53 mRNA in fibrosarcomas. We observed an increased frequency, although not significant, of mutant p53 in LnL compared to L group. We therefore focused on p53 wild type only, and we confirmed that the levels of p53 are higher in LnL than L mice (Figure 7B, t_(5)_ = 4.025, *p* = 0.0101). In the same mice expressing wild type p53, levels of p21 are not correlated to p53 levels and do not significantly differ between two groups (Figure 7C, t_(6)_ = 0.2492, n.s.).

Overall, these data support the hypothesis that delayed development of fibrosarcoma in the LnL group is due to increased activity of p53.

## 4. Discussion

Social environment can impact tumor progression in humans. In fact, while social isolation or events including divorce, loss of a close relative, or friend have been reported to predict high risk of cancer and poor cancer survival [22,23], psychosocial well-being and social support are protective factors against tumor development [1]. One French study assessed dispositional optimism in a group of 101 patients affected by head and neck cancer, and reported that life expectancy was longer in patients that were more optimistic compared to both more pessimistic patients and patients living alone and lacking social support [2]. Two other studies [5,6] were conducted on women treated for breast cancer and supported with psychological interventions aimed at improving well-being and mood. With respect to control (non-psychologically supported patients), women that received psychological support declared lower anxiety and distress, along with increased perception of social support. Furthermore, isolation of leukocytes from their peripheral blood revealed improved lymphocyte proliferation.

Despite this epidemiological evidence, few studies have directly investigated this issue and the underlying biological mechanisms remain largely unknown.

Using a combination of enrichment conditions and DNA damage-mediated tumor induction, we reported that exposing mice pups to early physical and social enrichment delays tumor progression in adulthood. Compared to mice raised in standard conditions, pups raised during the first postnatal four weeks by two females (LnL condition) are more reactive to environmental changes as they emit more USVs and keep higher body temperature during maternal absence. These same LnL mice display a slower development of fibrosarcomas induced by 3MCA injection in adulthood.

Environmental enrichment (EE) has been widely used in animal studies to assess the impact of positive environmental factors on different pathologies, including cancer. Several studies pointed out that in tumor-bearing mice, exposure to EE can reduce tumor growth and volume as well as improve the outcome of immunotherapy [24,25,26]. However, in EE, several factors are simultaneously acting, making it difficult to understand which factor is mainly responsible for the anti-tumor effects [27]. Furthermore, the above studies were performed with mice at adult ages under continuous EE stimulation.

To circumvent these limitations, we used the LnL condition that reproduces physical and social enrichment but is temporally restricted to early developmental stages. In fact, pups’ sensorimotor capabilities are limited during early development while their physiological plasticity is maximal. Hence, enrichment during infancy could result in long-term modifications. One other advantage in using the LnL condition is that the simultaneous presence of the biological mother plus a virgin non-lactating female increases physical and social stimuli without affecting the amount of breastfeeding, which may have long-term effects on the health system.

Previous studies using the same enrichment procedure reported that LnL condition can improve synaptic plasticity and cognitive functions and revert the autistic phenotype in mouse models of autism spectrum disorders [17,18,19]. Of note, in these studies, the positive effects of the LnL condition were evident during adulthood. However, the enrichment condition was only maintained during the first four postnatal weeks, meaning that LnL can have long-term effects due to persistent changes in biological systems. Indeed, developing biological systems in young pups are more responsive to environmental stimulation and more prone to stable modifications persisting in adults. Based on our previous studies, we set a quite large temporal gap (4 months) between the end of social enrichment condition and the 3MCA injection. The results confirmed the long-term protective effect of LnL condition towards fibrosarcoma development as well. These results are in line with a set of studies demonstrating that exposure to protective factors can drive persistent changes in molecular pathways, which are sufficient to trigger anti-tumoral responses upon carcinogenic insult [28,29]. Therefore, this model represents a stable condition, probably epigenetically induced, that does not require recall before the tumorigenic insult and could allow confinement of the altered molecular mechanisms for study.

In our model, molecularly associated with these results, we observed increased p53 activity in healthy tissue and decreased levels of Mdm2, the main inhibitor of p53. These data may suggest the potential involvement of this crucial oncosuppressor factor in the development of fibrosarcoma. Accordingly, we observed increased p53 protein levels in tumor tissue, supporting a potential increased oncosuppressive activity. Mirroring this, p53 was previously involved in stress response: chronic restraint causes decreased p53 function and promotes the growth of human xenograft tumors in a largely p53-dependent manner [15]. At present, it is not known what drives the increased p53 activation observed in our model. Indeed, many signalings contribute to activating p53 [9], and future studies will be required to dissect these pathways. Furthermore, we cannot exclude the fact that besides p53 activation, other factors also participate in the delayed onset of tumors found in our study. Among these factors, boosting of immune system and enhanced β-adrenergic signaling are also activated upon environmental stimulation [24,26]. Although our study excludes differences in T lymphocytes, further studies are needed to better explore the involvement of the immune system in our model.

Taken together, this study provides evidence that early social enrichment can antagonize DNA damage-induced tumorigenesis in the mouse with the possible involvement of p53 activity.

## Data Availability

Not applicable.

## Supplementary Materials

The following supporting information can be downloaded at: https://www.mdpi.com/article/10.3390/biom12040532/s1, Figure S1: LnL condition has no impact on basal lymphocytes levels assessed in 2 months old mice; Figure S2: Negative correlation between Mdm2 and p53; Figure S3: Unaltered Bax levels; Figure S4: P53 levels are increased in fibrosarcoma of LnL animals.
