# Supplementary material for: Early Social Enrichment Modulates Tumor Progression and p53 Expression in Adult Mice
Source: Biomolecules. 2022 Mar 31;12(4):532. doi: 10.3390/biom12040532 (PMC9032412; doi:10.3390/biom12040532)
Supplement: Supplementary file 1 [file biomolecules-12-00532-s001.zip › biomolecules-1654900-supplementary.pdf]

Supplementary Figures

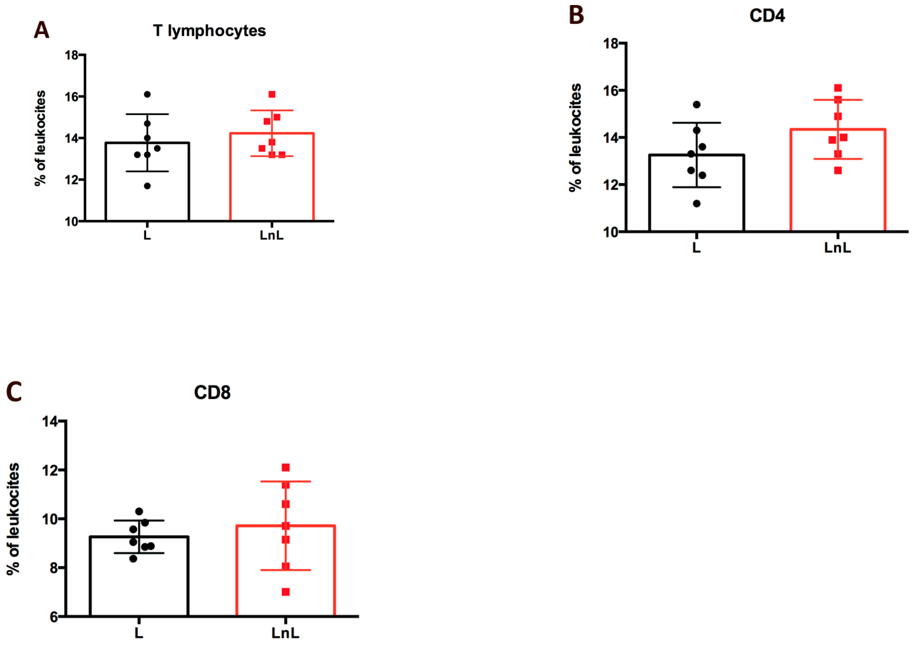

Figure S1. LnL condition has no impact on basal lymphocytes levels assessed in 2-months old mice. Graphs report the percentage of leukocytes (mean±SEM) classified as (**A**) T lymphocytes ($t_{(12)}$=0.686, p=0.505, n.s.), (**B**) CD4+ T lymphocytes ($t_{(12)}$=1.550, p=0.147, n.s.) and (**C**) CD8+ T lymphocytes ($t_{(12)}$=0.618, p=0.547, n.s.).

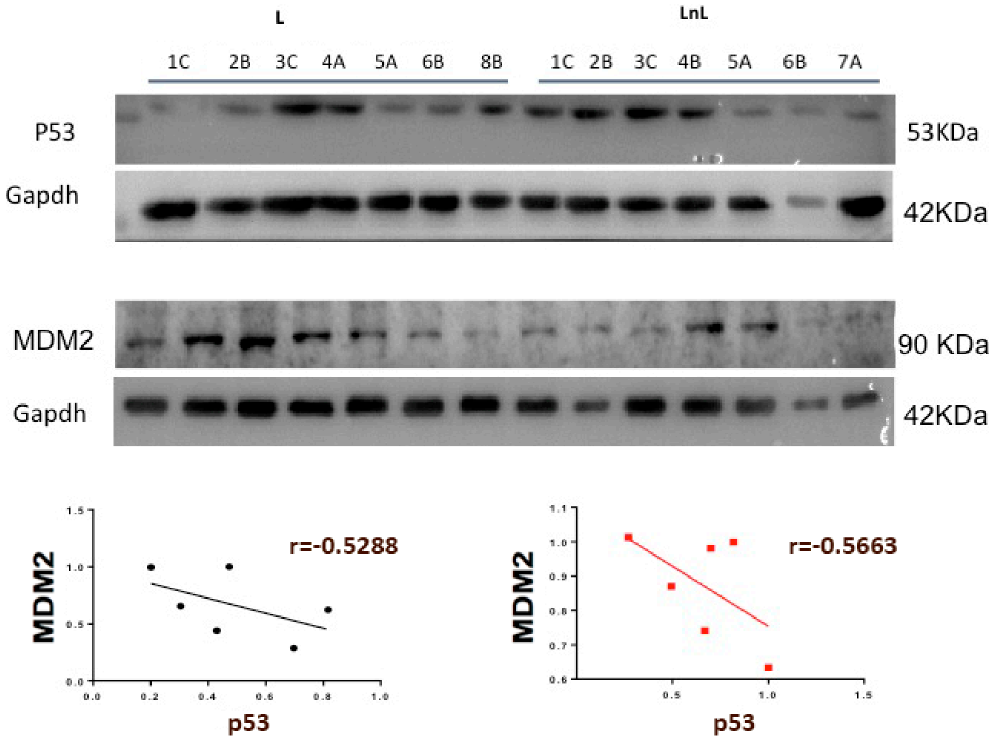

Figure S2. Negative correlation between Mdm2 and p53. (**Top**) WB analysis of indicated protein levels in muscles derived from animals of L and LnL groups, GAPDH was used as loading control. (**Bottom**) Graphs reporting a negative correlation between p53 and Mdm2 proteins levels in L and LNL groups. The relationships were compared between each pair of variables by Pearson correlation coefficient (confidence interval 95%), p-value of <0.05 considered as significant.

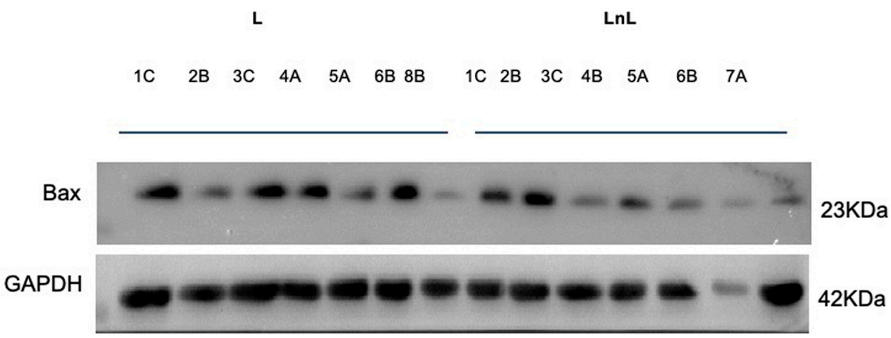

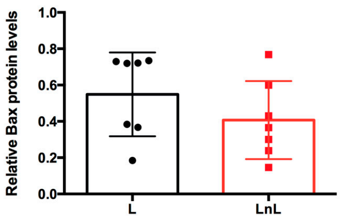

Figure S3. Unaltered Bax levels. (**Top**) WB analysis of indicated protein levels in muscles derived from animals of L and LnL groups, GAPDH was used as loading control. (**Bottom**) Grph reporting densitometric analysis of Bax relative to GAPDH levels (mean±SD) in indicated groups (two-tailed unpaired t-test: $t_{(12)}$=1.190, p=0.257, n.s.; n=7/group).

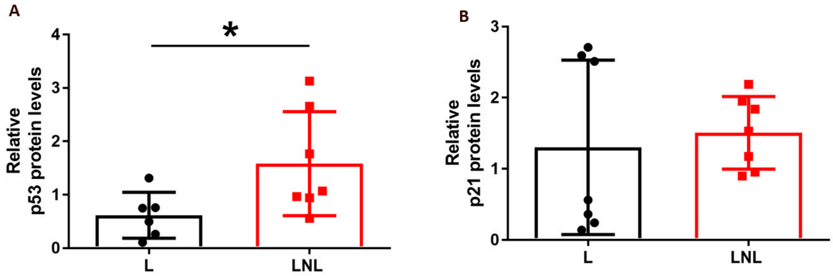

Figure S4. P53 levels are increased in fibrosarcoma of LnL animals. (**A**) Densitometric analysis of p53 relative to GAPDH levels (mean±SD) in indicated groups ($t_{(11)}$=2.247, p=0.0461, two-tailed unpaired t-test n=7, *p<0.05). (**B**) Densitometric analysis of p21 relative to GAPDH levels (mean±SD) in indicated groups (n.s., two-tailed unpaired t-test n=7). Note: these data include samples expressing both mutated and wild type p53 (see Figure 7).
